# Analysis and Assessment through Mechanical Static Compression Tests of Damping Capacity in a Series of Orthosis Plantar Materials Used as Supports

**DOI:** 10.3390/ijerph18010115

**Published:** 2020-12-26

**Authors:** Manuel Pabón-Carrasco, María Reina-Bueno, Samuel Vilar-Palomo, Inmaculada C. Palomo-Toucedo, Javier Ramos-Ortega, José María Juárez-Jiménez

**Affiliations:** 1 Department of Nursing, University of Seville, 41009 Seville, Spain; mpabon2@us.es (M.P.-C.); samuelvilarpalomo@hotmail.com (S.V.-P.); 2 Department of Podiatry, University of Seville, 41009 Seville, Spain; ipalomo@us.es (I.C.P.-T.); jrortega@us.es (J.R.-O.); jmjuarez@us.es (J.M.J.-J.)

**Keywords:** damping, biocompatible materials, polymers, foot, foot orthoses

## Abstract

High plantar pressure is the cause of multiple types of foot injuries and one of the main reasons for patient visits in podiatry and traumatology. Therefore, there is a need to acquire specific tools to address such injuries. The aim of this study was to determine the absorption capacity of selected materials applied as plantar supports and their response to pressure. The study had a cross-sectional design. A total of 21 materials were chosen and different material families were assessed, including ethylene-vinyl acetate, polyurethane foams, and polyethylene foams. Static compression tests were performed to analyze each material. The system is ideally suited for lower-force applications, small components, biomedical applications, and lower-strength materials. Damping was determined using mathematical calculations performed on the study data. It was found that materials with a low Shore A, or soft materials, exhibited worse absorption capacity than harder materials. Ethyl-vinyl acetates had good absorption capacity, polyurethane foams had a poor absorption capacity, and soft materials provided better adaption to impact. The results suggested that damping is not determined by the hardness of the material, and materials within the same family exhibit different damping capabilities.

## 1. Introduction

The feet are subjected to a series of loads resulting from their interaction with the ground [1]. At initial heel contact, the loads are hard and are sometimes regarded as impacts [2]. The elasticity of the feet enables them to support up to 200 kg of weight, thus providing efficient damping to avoid possible spinal injuries [1]. During normal gait, impacts tend to be equivalent to body weight. However, when running, jumping, or practicing different sports, these forces can duplicate or even multiply by factor of 10 depending on the activity [2].

Human beings possess a series of absorption mechanisms, such as the muscular action of the anterior tibial and the quadriceps and the subtalar eversion during the initial phase of the gait. Tendons and ligaments of the foot also absorb part of the energy of the shock, although the element of the foot which has, par excellence, a damping action is the plantar fat pad [3]. Nevertheless, these mechanisms are quite frequently not enough, and the use of plantar material supports is, therefore, required.

Plantar material supports feature elements for coping with plantar hyperpressure as they seek to redirect the forces that pass through the foot’s structure, avoiding abnormal compensator movements. To do so, they control the correct alignment of the forefoot and maintain the rearfoot in a neutral position. By increasing the area of contact, they achieve efficient redistribution of pressure between the foot, the orthosis, and its interaction with the ground. Plantar orthoses can be used to alleviate pain, increase damping, correct deformities, increase foot stability, and prevent skin injuries such as ulcers [4]. High plantar pressure is often the cause of these injures. This investigation focused on the concept of damping, which consists of utilizing a lower-density material to decrease the load on a specific point [2]. From a physical point of view, damping is the process that reduces load peaks and dissipates this energy by converting it into heat [5].

Most of the background focuses on the analysis of shoe materials or is related to the shoe industry. For example, Rodriguez-Perez (2001) studied diffusion in ethyl-vinyl acetates (EVA) foam under creep loading, concluding that the air content of the foam cells decreased, thus reducing cushioning [6]. Along the same line, Verdejo et al. assessed the mechanical interaction of the heel pad with running shoe midsoles, and they estimated the magnitude of internal heel pad stresses. The authors concluded that fatigue of the foam reduced heel strike cushioning [7]. On the other hand, Mills et al. evaluated polymer foams for personal protection including cushions, shoes, and helmets. The authors confirmed that with the flexible closed-cell foams used in shoe midsoles, cell air compression dominates the response. Diffusive air loss leads to foam deterioration with use. However, the authors themselves commented that the viscoelasticity of PU or EVA foams should also be considered [8]. Regarding the clinical problem related to the materials used in the plantar supports, commercial companies usually perform tests under unrealistic conditions, conducting studies using thicknesses greater than those used in the manufacture of plantar supports [4,9]. Additionally, there is some controversy in terms of damping. Some clinicians attribute cushioning properties to soft materials [4,10]. Therefore, this study makes it possible to clarify concepts from the engineering branch to the manufacture of plantar orthoses.

The purpose of this study was to assess the damping capacity of selected materials used in the fabrication of plantar orthoses, and to examine the correlation between hardness and damping capacity. Furthermore, the behavior after applying compression forces was evaluated.

## 2. Materials and Methods

The research design was cross-sectional and based on a trial performed in an engineering mechanics laboratory [11]. The final study sample comprised a total of 21 materials included in different families and combinations between them. The inclusion criteria were considered materials used in the manufacture of foot orthoses. The most representative thicknesses were those chosen in the manufacture of plantar orthoses, as well as the most common hardnesses. Materials used to reduce pressure on the feet of diabetic individuals, such as wool felts or liquid materials such as silicone, were excluded.

**Figure 1:**
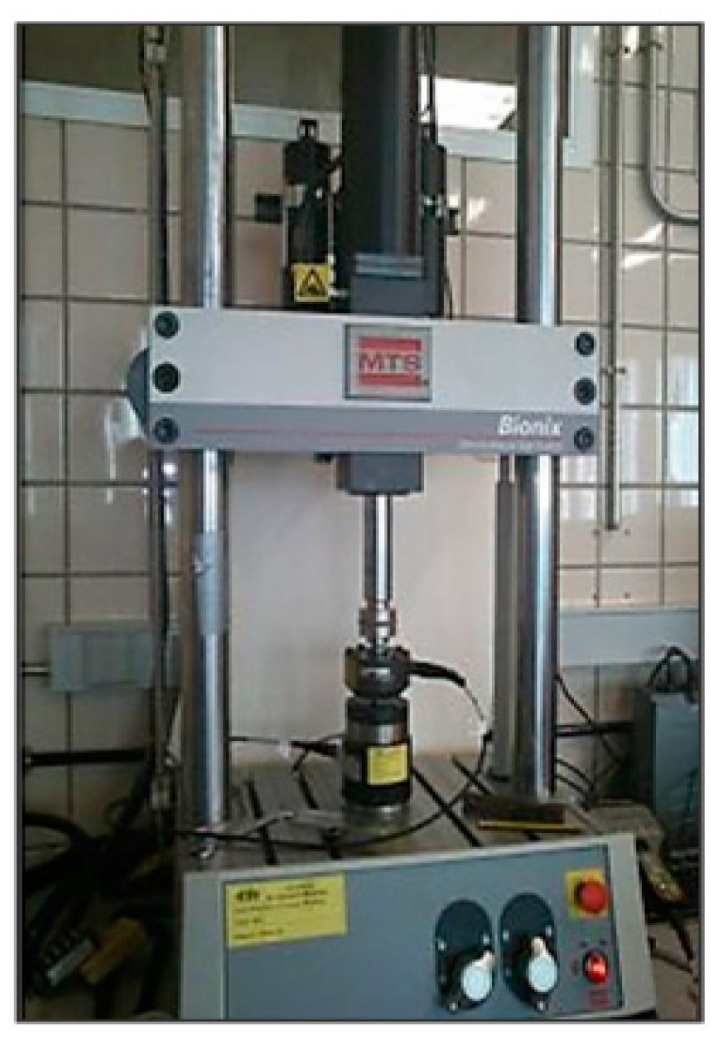
MTS Bionix 858^®^.

The MTS Bionix 858^®^ (MTS Systems Corporation, Eden Prairie, MN, USA) (Figure 1) test machine was used to evaluate damping capacity. The 858^®^ Testing System is often used to perform static and dynamic tests on materials with little resistance, such as plastics and some metals. Test engineers rely on Testing Systems (MTS) to achieve outstanding results for both static and dynamic material and component testing. In addition to being extremely compact, the 858 system provides a broad range of test enhancing features, including force ranges from 5 kN (1.1 kip) to 25 kN (5.5 kip), a moderate performance range, and the ability to test lower-strength materials, ranging from elastomers to aluminum. This device for material testing also accommodates specimens of different sizes (from sub-size to standard), and is capable of performing tension, compression, bend and fatigue tests, specialized tests for biomedical and biomechanical testing, and durability testing on small components.

Regarding the study, different pressures were applied (which had previously been obtained from patients) via pressure insoles featuring F-Scan^®^ sensors (Tekscan, South Boston, MA, USA). The pressures obtained within the footwear of the subjects was then evaluated. These pressures were transferred to the device, which applied the force, and the data were plotted to represent the loading and unloading of the material. Once the pressures were obtained, the test materials were fabricated so that they all had the same characteristics (same diameter) [12,13].

Pressure is defined as the ratio of force to surface area. Therefore, knowing the surface area and pressures, we can determine the forces exerted. A specimen with a radius of 2.75 cm was cut out, which makes it possible to calculate, using the area formula πr^2^, the surface of our test specimens. They all had the same contact surface. With the pressure values of the sensors, obtained from absolute rest to fast walking cadences, ranging between 0–3.5 kgf/cm^2^, the Newton (N) to be applied by the testing machine on each specimen was calculated using the following formula: gravity (9.8) × surface area × pressure kg/cm^2^ (0–3.5 kgf/cm^2^) (0–815 Newton).

**Figure 2:**
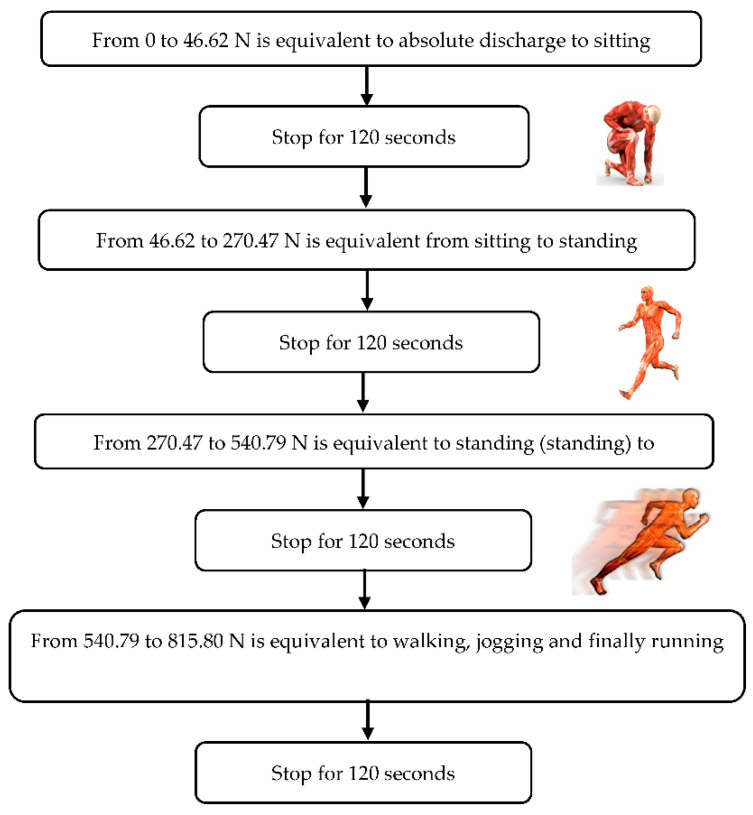
Work algorithm in statics.

Finally, the following data were entered into the program: the force to be applied, the number of cycles, and the duration of each cycle; tests were performed both statically and dynamically. The static study allowed us to assess the damping capacity of the materials (Figure 2).

**Figure 3:**
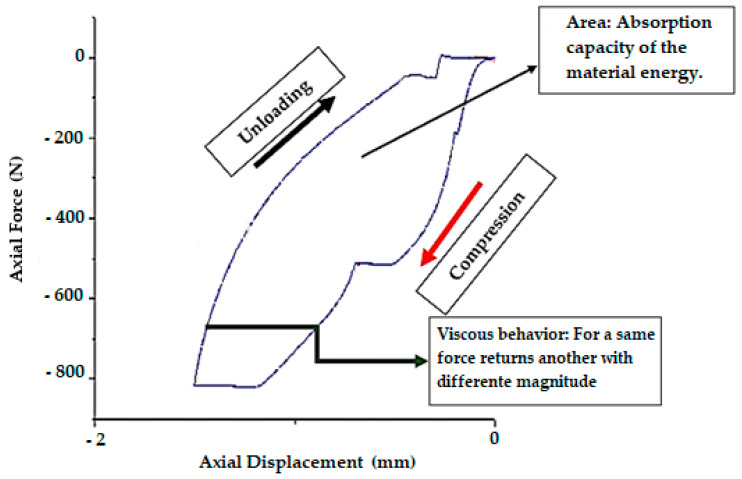
Representation of the material studied, in which the line of loading and unloading is apparent, leaving the energy dissipated between both (damping) (Static test).

More than 110,000 points were measured and presented in results graphs, which represent hysteresis curves. Damping was assessed by calculating the inside area of the hysteresis curves (via the cross-product method). This was obtained by calculating the area included between the two functions. This correlated with the energy absorbed by each material, as represented in Figure 3. For the same force another returns with a different magnitude.

As the hysteresis curves are described by closed polygonal lines, the shoelace formula was used to calculate the inside area (the generalization of the area of a triangle when the Cartesian coordinates of its vertices are known via the use of determinants) [14]. The aforementioned formula states that if the Cartesian coordinates (x, y) are the vertices of a closed polygon, the area of the enclosed polygon is [14]

$$A\left(P\right)=\frac{1}{2}\cdot I\left(\left(\Sigma^{i=n-1}x_{i}\cdot y_{i+1}-y_{i}\cdot x_{i+1}\right)\right)+(x_{n}\cdot y_{1-y_{n}}\cdot x_{1)}I$$

Statistical analysis was performed using SPSS version 18^®^ for Windows.

The Origin 8.0 program for Windows was used for the analysis and representation of data.

The Office 4.3 program and cross-product programming were used to calculate the area of regular polygons. A significance level of 0.05 was considered in all the hypothesis tests. Data exploration was performed to generate summary statistics for all cases. This procedure is used to identify atypical or extreme values and characterize differences between groups of cases. Likewise, it enabled us to identify whether the statistical techniques were appropriate and indicated the need to transform the data or use non-parametric tests [15].

First, the normality of observations for damping capacity (average of the dissipation of force of the materials) was assessed based on the Shapiro–Wilk test. If the normality assumption was satisfied, we first conducted the Levene test to check the equality of variance. When the normality assumption was not satisfied, we applied the Kruskal–Wallis test. When equality of variance was satisfied, the ANOVA test was performed [16].

The objective was to confirm whether there were relationships between the material’s hardness and its absorption capacity. The correlations between hardness and the dissipation area were evaluated using Pearson’s rank correlation coefficients [14].

## 3. Results

As mentioned in the Methods section, a total of 21 materials were studied (Table 1). Then, 21 tests were performed to evaluate static posture, 21 to evaluate dynamic posture, and 9 to evaluate wear and tear (Supplementary Material Figures S1–S7).

The thickness of the materials studied ranged between 3 mm and 6 mm. The most common thickness was 5 mm, and the average was 4.9 with SD 0.96.

The average hardness of the materials studied was between 29.81 and 40.98 Shore A, obtaining an overall average of 35.40 (SD 11.93) Shore A. The maximum/minimum values were 62 and 15.1 Shore A, respectively, and the confidence interval was 40.98–29.81.

The average dissipation of force of the materials studied was 532.21 ± 227.80 mm^2^, the maximum value was 886.55 mm^2^, and the minimum was 98.34 mm^2^ in this screening. The confidence interval was 642.01 to 422.41 mm^2^ (Table 1).

**Figure 4:**
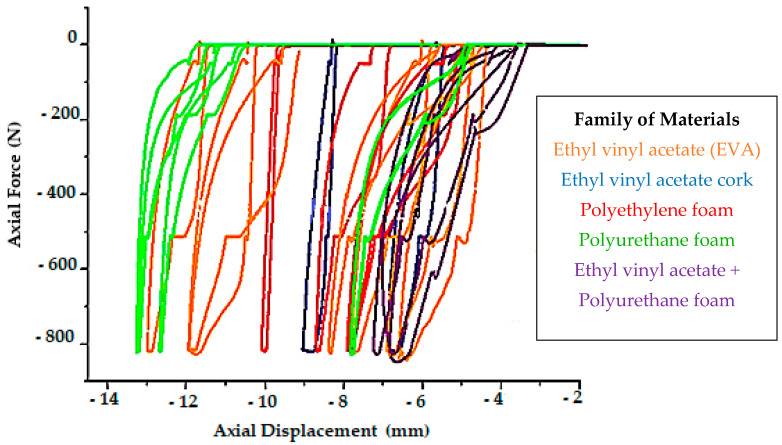
Representation of the materials subject to study, where the energy dissipation areas are apparent (Static test).

Graphs displaying the results were plotted for all the materials, as presented in Figure 4, and the hysteresis curves can be observed.

The Shapiro–Wilk normality test was used to verify whether the data followed a Gaussian distribution. The hardness (*p* = 0.059) and the dissipation area (*p* = 0.517) were obtained, and significant differences were observed in the damping capacity of the materials studied (*p* = 0.001). Taking these findings into account, the Pearson correlation test was applied to determine whether there was a relationship between the two variables studied (*p* = 0.507). It was observed that hardness had no relationship with the dissipation area of the forces of the materials studied, nor with reducing the material’s thickness and damping.

## 4. Discussion

The results of the energy dissipation area tests suggested that ethylene-vinyl acetates (EVAs) were the most effective materials (Table 1). However, the most interesting finding was that soft materials had a poor energy absorption capacity, thus displaying low hysteresis in the graph (Figure 2). This finding does not coincide with the traditional idea that they are good dampers [10].

The results demonstrated that polyurethane foams are not powerful dampers because they dissipate little energy. However, it must be highlighted that certain data showed them to be good pressure distributors and, as they adapt due to their foaminess and low hardness, they provide a fast response to impact. When pressure is exerted on a soft material, it rapidly deforms given that it cannot be depressed further, and it is highly rigid when there are moderate and high pressures, losing its capacity to dampen more energy. This finding is in accordance with the previous results from Expósito [17], who observed that lower-density EVAs were the material that was depressed most quickly. In contrast, harder EVAs can dampen more energy throughout the compression process because the material still retains the capacity to deform and to dissipate forces. In other words, EVAs are materials with greater hysteresis.

Polyurethane foams are materials that, given their fast adaptation to gait impact, must be placed in contact with the foot. This is a clinical point of view. From a practical point of view, materials such as foams achieve an increase in comfort due to their swift response, as it must not be forgotten that the gait cycle lasts approximately 1 s; thus, the foot needs the material to have this quality [18]. Studies on sports footwear with different compositions (e.g., ethylene-vinyl acetate, polyurethane, gel, air chambers) have not found significant relationships between the perception of comfort and biomechanical parameters. The only exception was in the case of Adidas shoes with Adiprene + ^®^ technology (high-density polyurethane), in which greater comfort and perception of damping was noted. Thus, Dinato et al. [19] concluded that it is quite complicated to predict the perception of comfort of a running shoe based on impact and plantar pressure.

In general, high-density materials are used to dampen, while low-density materials are useful in the redistribution of load, and those of medium density are more appropriate for shock absorption [20]. Taking into account the hardness of the material, high-density hard materials cannot store deformation energy and are, therefore, poor dampers [19,20]. Materials with a high capacity to store deformation and energy are ideal for the absorption of impacts. A material’s compression provides information about its capacity for deformation and rigidity [19,20]. Results of the present study support these previous observations with the exception that, due to the viscous nature of the materials, predicting their durability with compression tests is not exactly simple nor can it be easily validated.

Among the polyurethane foams studied in this investigation (Table 1), the one with the greatest absorption capacity was that of 18 Shore A and 5 mm thickness open-cell polyurethane. These findings coincide with the conclusions of Loy and Voloshin [21]. They observed that when jumping and going up and down stairs, forces were attenuated by the use of a viscoelastic elastomer under the heel, and that the degree of attenuation depended, to a great extent, on the thickness of the viscoelastic material used (8 mm was more efficient than 6 mm). Even et al. [22], who studied the viscoelastic properties of ethylene-vinyl acetate in the soles of sports footwear, noted that when the thickness of the sole in the heel decreased by 50%, the tension increased by 19%.

Thus, it can be deduced that impact absorption capacity is related to the thickness of the material. Yet, there are other important variables to take into account if we wish to dampen or want to increase propulsion. An open foamy structure has a high quantity of pores, thus enabling the air present to escape in each action of force and subsequently provide better adaptation to pressure. Closed cell polyurethane foams, as they do not allow air to escape, recuperate their thickness more quickly, increasing their elastic capacity (greater resilience). In fact, it is noted that the second polyurethane foam with a greater absorption of energy (Table 1) is not the second in terms of thickness, reaffirming the importance of the structure/density in the absorption of impacts (Table 1). According to the structure, two types of polyurethane foams are differentiated: some that have open cells exhibit rapid deformation of their structure, which absorbs greater energy (greater initial hysteresis and better adaptation to changes); and other polyurethane foams with closed cells (better resilience) are better suited to perform a propulsive function. Regarding the latter, this function implies that placing an elastic material under the foot means that it rebounds, thus producing an impulse that is again transferred to the foot. This means that the force is returned and, therefore, favors propulsion [22].

Open cell polyurethane foams may not be useful in patients whose professional activity obliges them to stand for a long time in a static foot posture. Essentially, as the material does not have a carryover of recuperation from active compression itself (as occurs in dynamic posture), it depresses without producing any effect because it is totally compressed. Moreover, as it does not help to pump blood, such conditions could cause foot fatigue.

The materials with the highest absorption capacity are EVAs, with 45 Shore A and 5 mm thickness, which feature an energy dissipation area of 886.55 mm^2^, followed by ethylene-vinyl acetate with 37 Shore A and the EVAs with 41 Shore A, 4 mm thickness (Table 1). It can be observed among the EVAs that those with greater hardness are the same ones that absorb more energy, contrary to the widespread belief that soft materials have a greater absorption capacity. As they have a high energy absorption capacity, these materials must be placed under polyurethane foams or low-density material, given that they absorb the impact transmitted by the foot against the ground, thus minimizing the inverse force of Newton’s third law, or law of action–reaction [23]. Following what is presented about hardness, it is evident that the EVAs 15 Shore A 5 mm, a material called “soft”, has a poorer damping capacity; however, due to its low Shore A, its spreading of pressures is more homogeneous, being comfortable for the person. Therefore, we emphasize that the hardness determines the order in the orthosis not because of its absorption capacity but due to its adaptation to impact(s).

Cheung et al. [24] studied factors that further alleviate foot pressure. Among the five factors considered in the design of their study—arch type, material of the insole, thickness of the insole, material of the sole, and inter-sole thickness—it was found that the use of a customized orthosis, which provides support to the arch and a soft material for the insole, were the two most effective factors to reduce the peaks of plantar pressures [25]. Cheung et al. [24] suggested that the insole layer should have a hardness value below 20° Shore A, with a view to maximizing its capacity to alleviate pressure. Notwithstanding, it must be taken into account that the hardness will depend on the pressure experienced in the insole, which will be determined by body weight and activity performed by the user.

Lane et al. [26] demonstrated that as the hardness of the footwear increases, the plantar pressures increase, but this does not appear to have a significant effect on the footwear’s comfortability. That study supports the results of our work, which infers that soft materials must be in contact with the foot given that, as they adapt more rapidly to its morphology, they distribute pressures more appropriately.

Currently, a multitude of plantar orthoses are used to compensate for different pathologies related to plantar hyper-pressure. Therefore, knowing the behavior of the materials from which the plantar orthoses are fabricated, which can be derived from this study, can be the “first stone” in the search to improve orthotic treatment.

**Figure 5:**
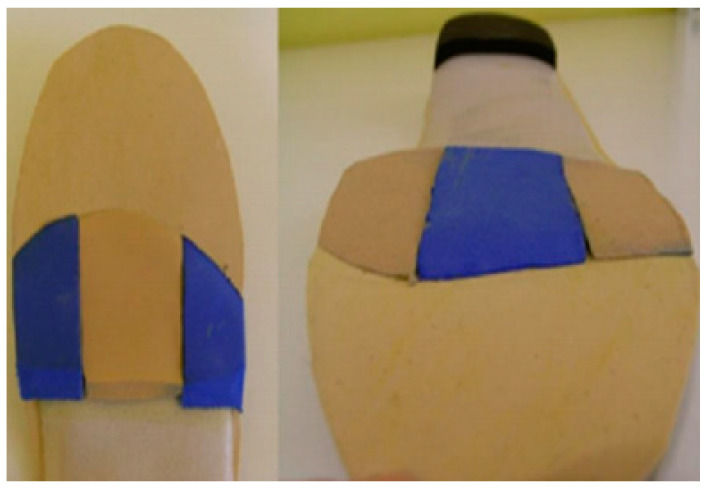
Metatarsal pads with polyurethane foams.

Finally, this study makes it possible to determine the order of materials based on their compression deformation capacity. Soft materials must be in contact with the foot for their speed of adaptation to impact [27]. For example, using metatarsal bars made from soft materials under a hard material would not make sense given the results of this work (Figure 5). This concept is more developed in the footwear industry, where the layers of the shoe are made based on density, hardness, capacity of damping, and deformation of the material. [28,29] On the other hand, it is not useful to use soft materials under harder materials. This is a common practice in the manufacture of foot orthoses. Added to the fact that the materials have a viscoelastic behavior, viscoelastic materials are not linear and depend on variables such as time of use [30].

The present study had some limitations. It should be taken into account that the tests were carried out under laboratory conditions. Although the conditions referring to gait cadences and pressures exerted during walking were reproduced, other factors, such as terrain variability, footwear, and biomechanics, were not considered. Therefore, the results should be assessed clinically. In addition, the fracture of the materials was not assessed.

## 5. Conclusions

This study concluded that hardness does not determine absorption capacity. However, it is useful to know which materials adapt more quickly to impacts. The materials have a viscoelastic behavior. There is no relationship between the loss of thickness and the damping capacity. The reduction in thickness is more related to adaptation to impact because it depends on the hardness of the material (ability to resist being penetrated).

Materials with a lower hardness have less absorption capacity, but they are useful to adapt the impact of the foot against the ground. These must be placed in contact with the foot and those of greater hardness and absorption capacity in contact with the ground.

On the other hand, some polyurethane foams are suitable to reduce impact, not because they cushion well over time, but because of their rapid adaptation to pressure changes, giving the patient comfort. EVAs are shown to be great dampers displacing polyurethane foams in second place.

## Figures and Tables

**Table 1:** Dissipation area of the different materials studied related with their thickness.

Materials	Thickness (mm)	Hardness (Shore A)	Thickness Reduction (mm)	Energy Dissipation Area (mm^2^)
Ethylene vinyl acetate	5 mm	45	2.34	886.55
Ethylene vinyl acetate	5 mm	37	2.85	870.13
Ethylene vinyl acetate	4 mm	41	1.78	762.78
Double layer EVA/Polyurethane	6 mm	39.4	3.45	757.05
Polyethylene foam Roval foam^®^	5 mm	36.5	3.16	739.72
Ethylene vinyl acetate	5 mm	39.1	2.19	728.32
Ethylene vinyl acetate	4 mm	30	2.69	664.49
Ethylene vinyl acetate	4 mm	40	2.05	646.24
Double layer EVA/Polyurethane	6 mm	32.5	3.27	584.20
Polyethylene foam Roval foam^®^	5 mm	39	3.10	559.76
Polyethylene foam Roval foam^®^	3 mm	36	1.95	553.69
Ethylene vinyl acetate	5 mm	15.1	3.95	518.543
Cork EVA	4 mm	41	1.52	510.50
Ethylene vinyl acetate	3 mm	39.4	1.58	485.40
Open-cell polyurethane	5 mm	18	3.17	387.09
Open-cell polyurethane	3 mm	16	1.82	322.23
Latex	5 mm	14	3.85	306.06
Cork EVA	5 mm	49	0.76	280.40
Closed-cell polyurethane	4 mm	22	2.29	220.76
Closed-cell polyurethane	3.2 mm	20	2.21	200.82
Polyethylene foam Roval foam ^®^	5 mm	62	0.52	98.43

## Data Availability

The data presented in this study are available on request of the authors.

## Supplementary Materials

The following are available online at https://www.mdpi.com/1660-4601/18/1/115/s1, Figure S1: Dynamics test: breakdown into gait cadence, Figure S2: Family: Polethylene foam Roval foam®. Analysis of the materials: Test in statics and test in dynamics, Figure S3: Family: Cork EVA. Analysis of the materials: Test in statics and test in dynamics, Figure S4: Family: Polyurethane. Analysis of the materials: Test in statics and test in dynamics, Figure S5: Family: Latex. Analysis of the materials: Test in statics and test in dynamics, Figure S6: Family: Ethylene vinyl acetate. Analysis of the materials: Test in statics and test in dynamics, Figure S7: Family: Double layer EVA/Polyurethane. Analysis of the materials: Test in statics and test in dynamics.
