# Supplementary material for: Analysis and Assessment through Mechanical Static Compression Tests of Damping Capacity in a Series of Orthosis Plantar Materials Used as Supports
Source: Int J Environ Res Public Health. 2020 Dec 26;18(1):115. doi: 10.3390/ijerph18010115 (PMC7794760; doi:10.3390/ijerph18010115)
Supplement: Supplementary file 1 [file ijerph-18-00115-s001.pdf]

Supplementary Materials

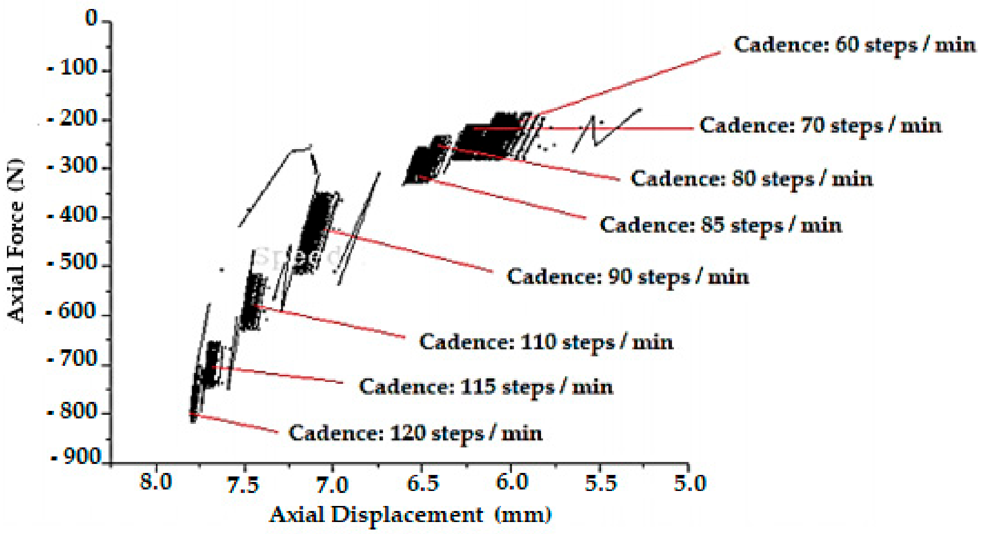

Supplementary Figure S1. Dynamics test: breakdown into gait cadence. Work algorithm in dynamics: 20 min 60 cycles 1 Hz 1200 cycles (186.5–279.7 N); 40 min 70 cycles 1.17 Hz 2800 cycles (209.8–279.7 N); 10 min 80 cycles 1.33 Hz 800 cycles (233.1–326.3 N); 12 min 85 cycles 1.42 Hz 1020 cycles (256.4–340.7 N); 8 min 90 cycles 1.5 Hz 720 cycles (349.8–512.8 N); 5 min 110 cycles 1.83 Hz 550 cycles (512.5–629.7 N); 5 min 115 cycles 1.92 Hz 575 cycles (652.6–745.9 N); 2 min 120 cycles 2 Hz 240 cycles (699.3–815.8 N); Overall: 102 min = 2709 MET * 7905 Cycles; MET * = MET is the unit of measure of the metabolic rate and is defined as the amount of heat emitted by a person in a sitting position per square meter of skin.

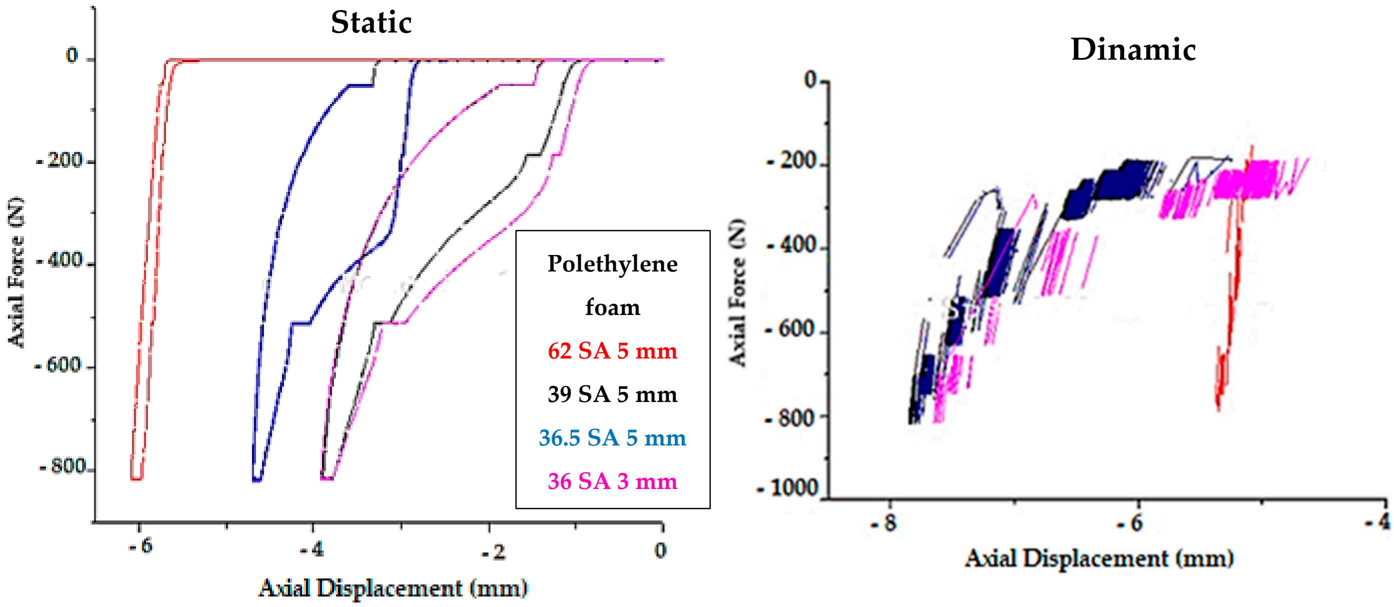

Supplementary Figure S2. Family: Polethylene foam (Roval foam®). Analysis of the materials: Test in statics and test in dynamics.

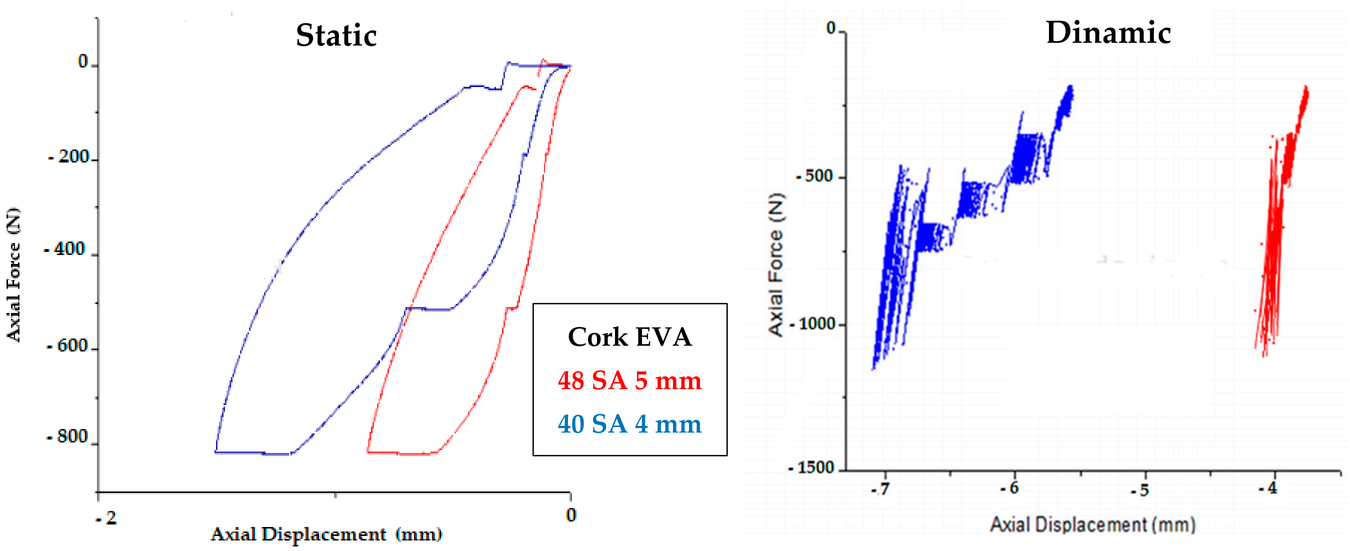

Supplementary Figure S3. Family: Cork EVA. Analysis of the materials: Test in statics and test in dynamics.

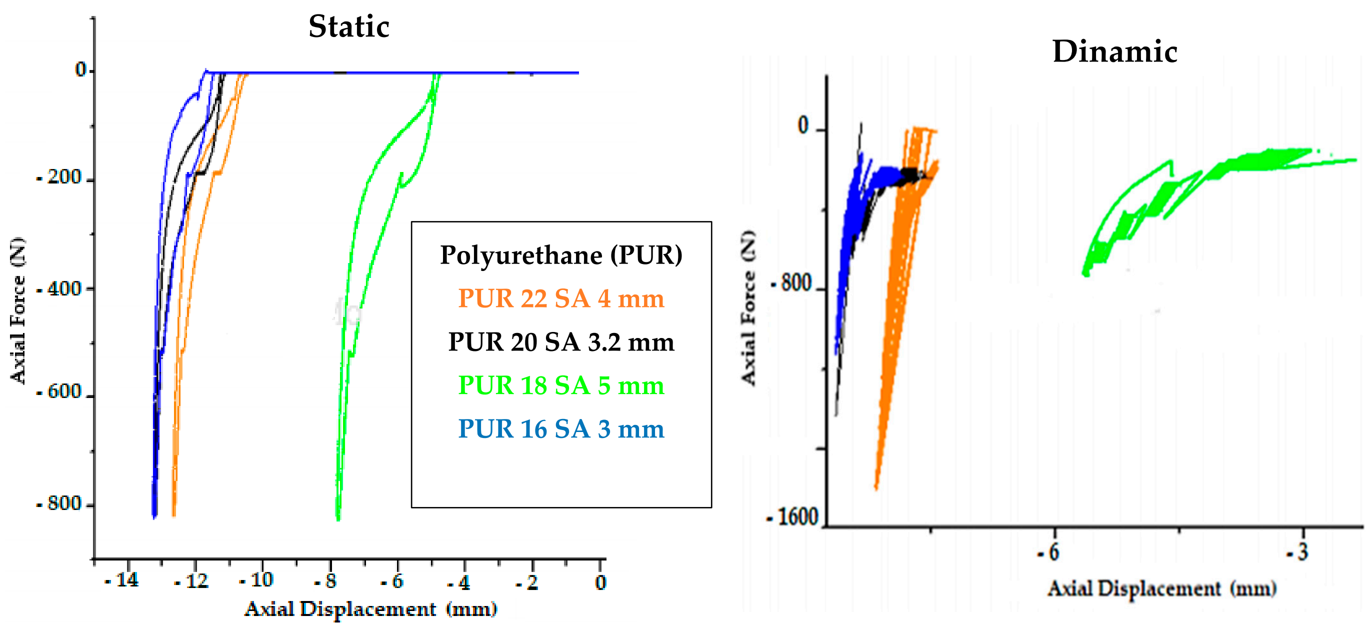

Supplementary Figure S4. Family: Polyurethane. Analysis of the materials: Test in statics and test in dynamics.

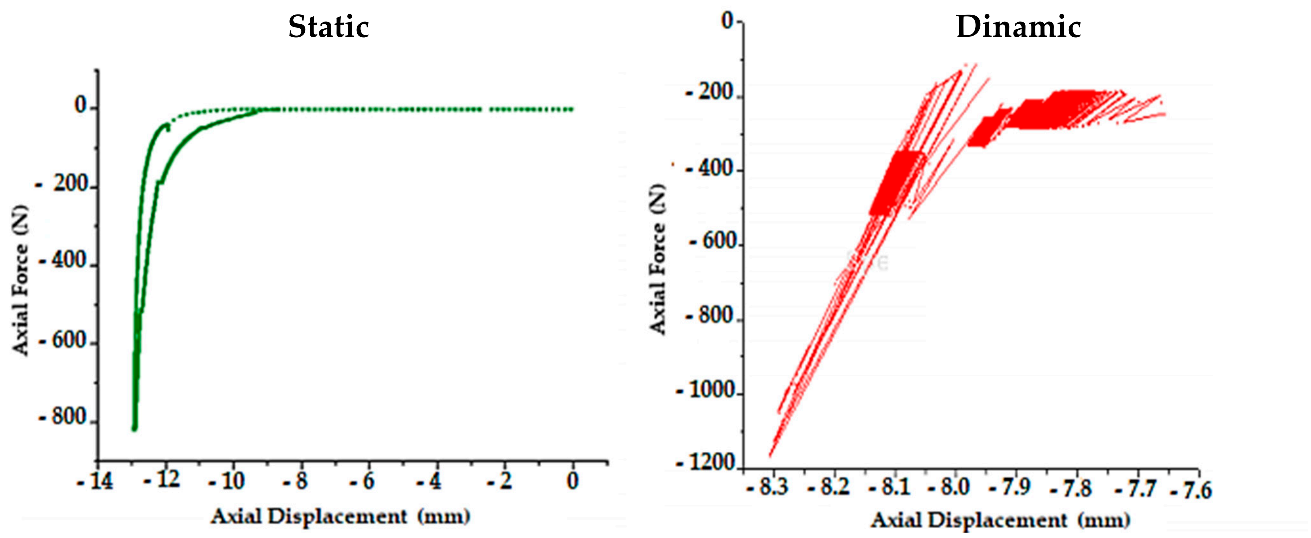

Supplementary Figure S5. Family: Latex. Analysis of the materials: Test in statics and test in dynamics.

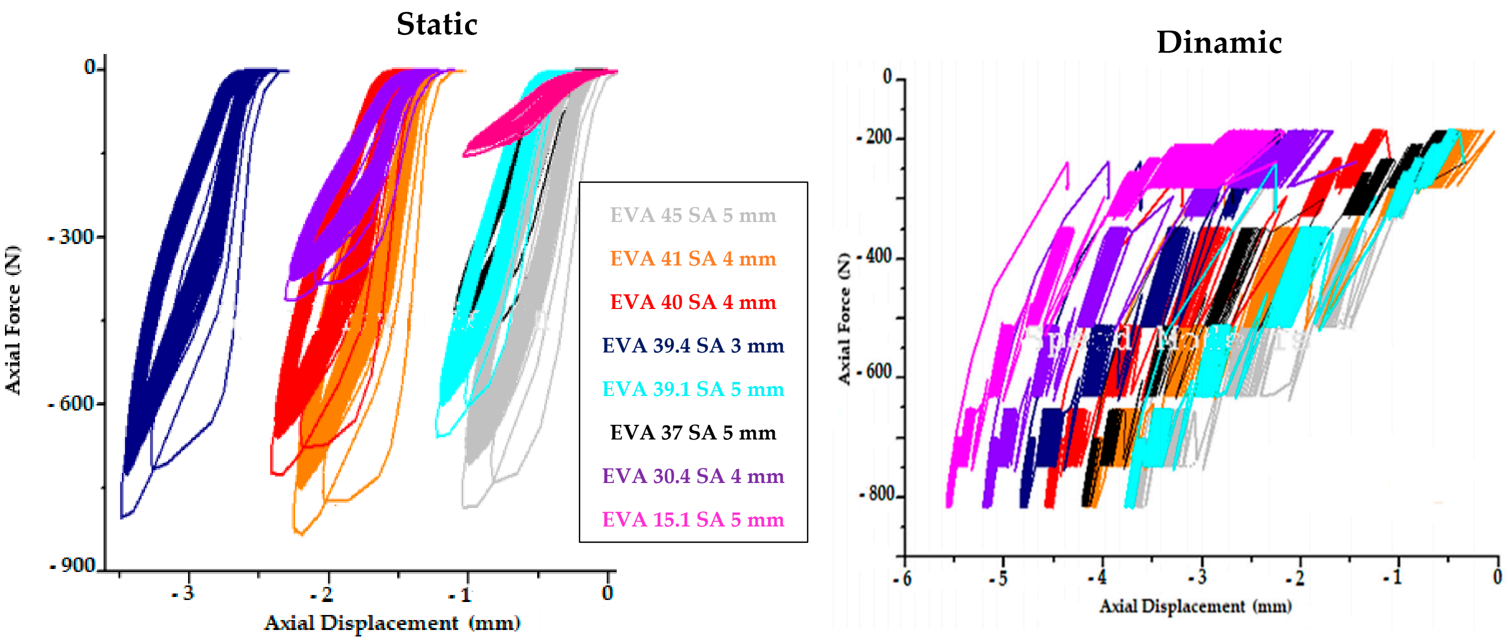

Supplementary Figure S6. Family: Ethylene vinyl acetate. Analysis of the materials: Test in statics and test in dynamics.

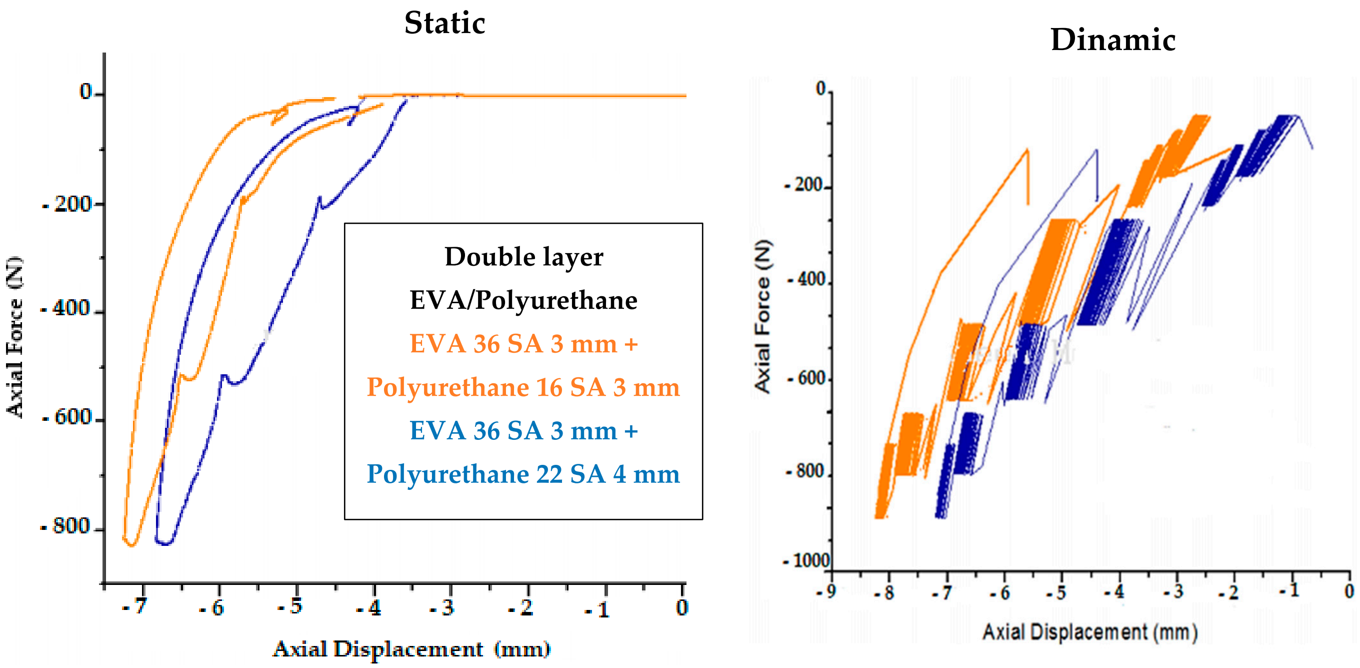

Supplementary Figure S7. Family: Double layer EVA/Polyurethane. Analysis of the materials: Test in statics and test in dynamics.
